# Innovative Strategies to Overcome Biofilm Resistance

**DOI:** 10.1155/2013/150653

**Published:** 2012-12-23

**Authors:** Aleksandra Taraszkiewicz, Grzegorz Fila, Mariusz Grinholc, Joanna Nakonieczna

**Affiliations:** Laboratory of Molecular Diagnostics, Department of Biotechnology, Intercollegiate Faculty of Biotechnology, University of Gdansk and Medical University of Gdansk, Kladki 24, 80-822 Gdansk, Poland

## Abstract

We review the recent literature concerning the efficiency of antimicrobial photodynamic inactivation toward various microbial species in planktonic and biofilm cultures. The review is mainly focused on biofilm-growing microrganisms because this form of growth poses a threat to chronically infected or immunocompromised patients and is difficult to eradicate from medical devices. We discuss the biofilm formation process and mechanisms of its increased resistance to various antimicrobials. We present, based on data in the literature, strategies for overcoming the problem of biofilm resistance. Factors that have potential for use in increasing the efficiency of the killing of biofilm-forming bacteria include plant extracts, enzymes that disturb the biofilm structure, and other nonenzymatic molecules. We propose combining antimicrobial photodynamic therapy with various antimicrobial and antibiofilm approaches to obtain a synergistic effect to permit efficient microbial growth control at low photosensitizer doses.

## 1. Introduction

Photodynamic therapy dates to the time of the pharaohs and ancient Romans and Greeks, for whom the connection between the sun and health was obvious. Until the 19th century, heliotherapy was the only known form of phototherapy [1]. Heliotherapy was used in thermal stations to cure tuberculosis and to treat ulcers or other skin diseases [2]. The 20th century brought significant developments in phototherapy, particularly in photodynamic therapy (PDT) directed against cancer as well as photodynamic inactivation (PDI) of microorganisms, also known as antimicrobial PDT (APDT). PDT has gained clinical acceptance, and many clinical trials are being conducted, while APDT is in its infancy. As antibiotic therapies become less effective because of increasing microbial resistance to antibiotics, alternative methods such as APDT for fighting infectious diseases are urgently needed. Microbial biofilms cause a large number of chronic infections that are not susceptible to traditional antibiotic treatment [3, 4]. Biofilm-forming microbes are held together by a self-produced matrix that consists of polysaccharides, proteins and extracellular DNA [5, 6].

## 2. Biofilm: Structure, Biology, and Treatment ****Problems

A microbial biofilm is defined as a structured community of bacterial cells enclosed in a self-produced polymeric matrix that is adherent to an inert or living surface [4, 7]. The matrix contains polysaccharides, proteins, and extracellular microbial DNA, and the biofilm can consist of one or more microbial (bacterial or fungal) species [5, 8]. The matrix is important because it provides structural stability and protection to the biofilm against adverse environmental conditions, for example, host immunological system and antimicrobial agents [6, 9]. Biofilm-growing microorganisms cause chronic infections which share clinical characteristics, like persistent inflammation and tissue damage [3]. A large number of chronic bacterial infections involve bacterial biofilms, making these infections very difficult to be eradicated by conventional antibiotic therapy [4]. Biofilm formation also causes a multitude of problems in the medical field, particularly in association with prosthetic devices such as indwelling catheters and endotracheal tubes [10]. Biofilms can form on inanimate surface materials such as the inert surfaces of medical devices, catheters, and contact lenses or living tissues, as in endocardium, wounds, and the epithelium of the lungs, particularly in cystic fibrosis patients [8, 11, 12]. Microbial antigens stimulate the production of antibodies, which cannot effectively kill bacteria within the biofilm and may cause immune complex damage to surrounding tissues [13]. Regardless of the presence of excellent cellular and humoral immune reactions, host defense mechanisms are rarely able to resolve biofilm infections [14]. The symptoms caused by the release of planktonic cells from the biofilm can be treated by antibiotic therapy, but the biofilm remains unaffected [15]. Thus, biofilm infection symptoms are recurrent even after several antibiotic therapy cycles, and the only effective means of eradicating the cause of the infection is the removal of the implanted device or the surgical removal of the biofilm that has formed on live tissue [16]. Biofilm-growing bacteria differ from planktonic bacteria with respect to their genetic and biochemical properties. Biofilm-forming bacteria coaggregate with each other and with multiple partners and form coordinated groups attached to an inert or living surface; they surround themselves with polymer matrix, communicate effectively via quorum sensing mechanisms, and express low metabolic activity limiting the impact of conventional antimicrobials acting against actively metabolizing cells [4, 7, 12].

### 2.1. Biofilm Formation

Biofilm formation can be divided into three main stages: early, intermediate, and mature [17]. During the early stage, planktonic cells swim along the surface often using their flagella mode of movement or they can be transferred passively with the body fluids (Figure 2). Next, the contact between microorganisms and a surface is made, resulting in the formation of a monolayer of cells [18–20]. At this stage, the bacteria are still susceptible to antibiotics, and perioperative antibiotic prophylaxis can be critical for successful treatment [6, 9]. The importance of the first attachment step was confirmed by experiments with surface attachment-defective (sad) mutant strains of *Pseudomonas aeruginosa*, which are unable to form biofilms [21]. The next step involves irreversible binding to the surface, multiplication of the microorganisms, and the formation of microcolonies [6, 9]. During this stage, the polymer matrix is produced around the microcolonies and generally consists of a mixture of polymeric compounds, primarily polysaccharides (the matrix contributes 50%–90% of the organic matter in biofilms) [22, 23]. Studies on *Candida albicans* have demonstrated that during the third stage (the maturation phase), the amount of extracellular material increases with incubation time until the yeast communities are completely encased within the material [17]. The matrix consists mainly of water, which can be bounded within the capsules of microbial cells or can exist as a solvent [24]. Apart from water and microbial cells, the biofilm matrix is a very complex material. The biofilm matrix consists of polymers secreted by microorganisms within the biofilm, absorbed nutrients and metabolites, and cell lysis products; therefore, all major classes of macromolecules (proteins, polysaccharides, and nucleic acids) are present in addition to peptidoglycan, lipids, phospholipids, and other cell components [25–27]. The third step of biofilm formation is the formation of a mature community with mushroom-shaped microcolonies [3]. During this stage, the biofilm structure can be disrupted, and microbial cells can be liberated and transferred onto another location/surface, causing expansion of the infection [6, 9].

 Biofilm formation is regulated at different stages through diverse mechanisms, among which the best studied is quorum sensing (QS) [28–31]. The QS mechanism involves the production, release, and detection of chemical signaling molecules, which permit communication between microbial cells. The QS process regulates gene expression in a cell-density-dependent manner; for biofilm production, the genes involved in biofilm formation and maturation are activated at a critical population density [32–34]. There are three well-defined groups of signaling QS molecules in bacteria: oligopeptides, acyl homoserine lactones (AHLs), and autoinducer-2 (AI-2) [34]. Gram-positive bacteria predominately use oligopeptides as a communication molecule, and AHLs are specific for Gram-negative bacteria [35, 36]. AI-2 is reported to be a universal signaling molecule that is used for both interspecies and intraspecies communication [34]. Boles and Horswill proposed that the *Staphylococcus aureus agr* quorum sensing system controls not only the switch between planktonic and biofilm growth but also the mechanism of the dispersal of cells from an established biofilm [37]. Moreover, results from our research group indicate that *agr* polymorphism could impact biofilm formation and directly influence bacterial susceptibility to photoinactivation (data not shown). 

### 2.2. Biofilm Resistance

Infections caused by biofilm-forming bacteria are often difficult to treat. Biofilm formation almost always leads to a large increase in resistance to antimicrobial agents (up to 1000-fold decrease in susceptibility) in comparison with planktonic cultures grown in conventional liquid media [4, 7]. A few mechanisms of biofilm resistance to antibiotics have been proposed. The first proposed mechanism involves the matrix, which represents a physical and chemical barrier to antibiotics. Ciofu et al. [38] demonstrated that the resistance of *P. aeruginosa* biofilms to antimicrobial treatment is related to mucoidy. Mucoid biofilms were up to 1000 times more resistant to tobramycin than nonmucoid biofilms, in spite of similar planktonic MICs [38]. Anderl et al. demonstrated that ciprofloxacin and chloride ion could penetrate a wild-type *Klebsiella pneumoniae* biofilm, while ampicillin could not [39]. By contrast, ampicillin rapidly penetrated a *β*-lactamase-deficient *K. pneumoniae* biofilm. The authors assumed that the biofilm matrix was not an inherent mechanical barrier to solute mobility and that ampicillin failed to penetrate the biofilm because it was deactivated by the wild-type biofilm at a faster rate than it could diffuse into the film [40]. Jefferson et al. suggested that even though the matrix may not inhibit the penetration of antibiotics, it may retard the rate of penetration enough to induce the expression of genes within the biofilm that mediates resistance [41]. A second hypothesis to explain reduced biofilm susceptibility to antibiotics concerns the metabolic state of microorganisms in a biofilm. Some of the cells located deep inside the biofilm structure experience nutrient limitation and therefore exist in a slow-growing or starved state [42]. Nutrient-depleted zones within the biofilm can result in a stationary phase-like dormancy that may influence the general resistance of biofilms to antibiotics. Walters et al. demonstrated that oxygen penetrated from 50 to 90 *μ*m into colony biofilms formed by *P. aeruginosa* and that the antibiotic action is focused near the air-biofilm interface [43]. This study also showed that oxygen limitation has a role in antibiotic resistance [43]. Slow-growing or nongrowing cells are not very susceptible to many antimicrobial agents because the cells divide infrequently and antibiotics that are active against dividing cells (such as beta-lactams) are not effective. The third hypothesis involves genetic adaptation to different conditions. The mutation frequency of a biofilm-growing microorganism is significantly higher than that of its planktonic form; for *P. aeruginosa*, up to a 105-fold increase in mutability has been observed [44]. A recent study by Ma and Bryers demonstrated that donor populations in biofilms (containing a plasmid with a kanamycin resistance gene) exposed to a sublethal dose of kanamycin exhibited an up to tenfold enhancement in the transfer efficiency of the plasmid [45]. At least some of the cells in a biofilm are likely to adopt a distinct phenotype that is not a response to nutrient limitation but a biologically programmed response to growth on a surface [4, 7]. Several genes are involved in biofilm formation and some of the genes are exclusively expressed in biofilm-growing microorganisms [46, 47].

All published results indicate that a reduction in the efficiency of photodynamic treatment occurs when PDI is applied to biofilm-related experimental models. Thus, it is necessary to identify factors that disrupt biofilm structure or affect biofilm formation.

## 3. Antimicrobial Photodynamic Therapy

**Figure 1:**
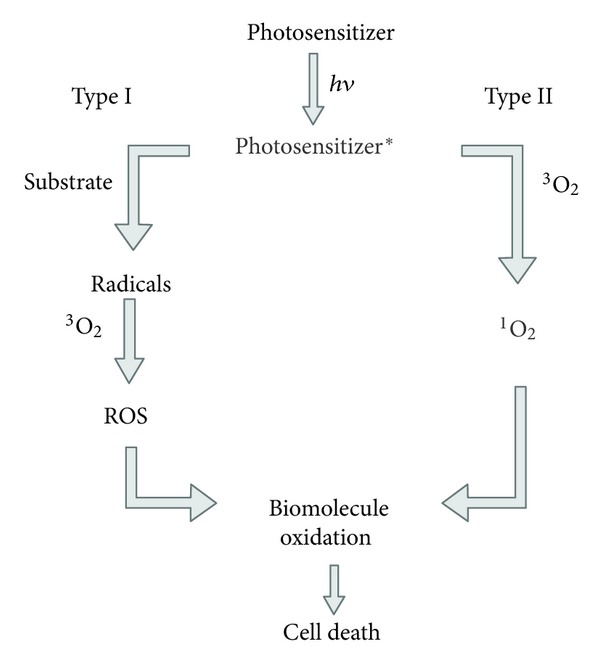
Scheme of photodynamic processes. Photosensitizer in excited state forms a long-lived triplet excited state. Type I reactions involve electron transfer from the triplet-state PS to a substrate, leading to production of, for example, lipid-derived radicals which can combine or react with other biomolecules and oxygen, eventually producing reactive oxygen species. In type II reactions, the energy is transferred from the triplet state PS to a ground state (triplet) molecular oxygen to produce excited singlet-state oxygen which can oxidize biomolecules in the cell. Both forms of reactive oxygen can cause cell damage and death.

**Figure 2:**
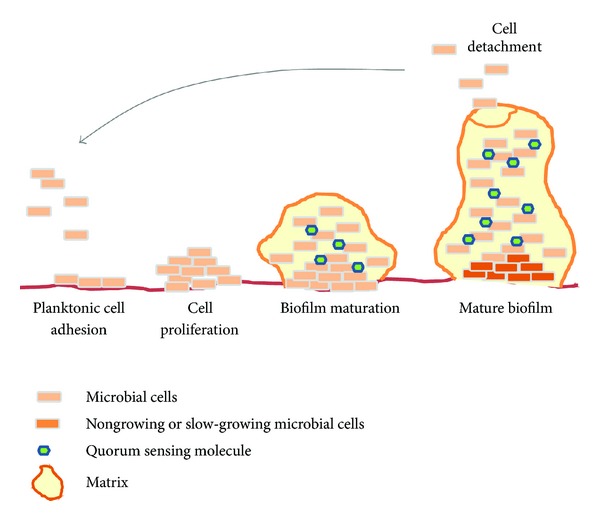
Biofilm formation. Planktonic cells adhere to the surface and proliferate. During biofilm maturation, the extracellular matrix and quorum sensing molecules are produced. Mature biofilm is characterized by a large number of matrices, slow-growing microbial cells in the center, and fragmentation which leads to cell detachment and spread of infection.

Photodynamic therapy consists of three major components: light, a chemical molecule known as a photosensitizer, and oxygen. The photosensitizer (PS) can be excited by absorbing a certain amount of energy from the light. The excitation occurs when the wavelength range of the light overlaps with absorbance spectrum of the photosensitizer. After excitation, photosensitizers usually form a long-lived triplet-excited state, from which energy can be transferred to biomolecules or directly to molecular oxygen, depending on the reaction type (Figure 1). Type I (Figure 1) reactions involve electron transfer from the triplet state PS to a substrate, for example, unsaturated membrane phospholipids or aminolipids, leading to the production of lipid-derived radicals or hydroxyl radicals (HO^∙^) derived from water. These radicals can combine or react with other biomolecules and oxygen to yield hydrogen peroxide, causing lipid peroxidation or leading to the production of reactive oxygen species that can cause cellular damage and cell death [48]. Type II (Figure 1) reactions involve energy transfer from the triplet-state PS to ground-state (triplet) molecular oxygen to produce excited singlet-state oxygen, which is a very reactive species with the ability to oxidize biomolecules in the cell such as proteins, nucleic acid, and lipids, causing cell damage and death [48]. Both mechanisms can operate in the cell simultaneously, but type II is generally considered the major APDT pathway [49]. There are two major types of cellular damage: DNA damage and the destruction of cellular membranes and organelles. Because the cell is protected by DNA repair systems, DNA damage may not be the main cause of microbial cell death. A large portion of the microbicidal effect of APDT may be due to the disruption of proteins involved in transport and membrane structure and the leakage of cellular contents [49].

Recent studies have shown that the antimicrobial effect can be obtained with the use of photosensitizers belonging to different chemical groups. The most studied PSs are phenothiazine dyes (methylene blue (MB) and toluidine blue O (TBO)), porphyrin and its derivatives, fullerenes, and cyanines and its derivatives (Table 1). More studies have been conducted of forms of microbial growth other than planktonic growth (Table 2). The problem of several chronic microbial infections is now known to be inseparable from biofilm formation by pathogens. Thus, *in vitro* studies have concentrated more on biofilm models as well as *in vivo* models, particularly rat and mouse models of infected wounds (Table 2). As we have discussed previously, when studying the use of photoinactivation in biofilm-related models, the mechanism of strain-dependent response to PDI requires further investigation [50–53].

### 3.1. Recent *In Vitro* Studies

#### 3.1.1. Planktonic Culture of Microorganisms

In *in vitro* studies of phenothiazine dyes, Lin et al. demonstrated that MB can be successfully used to eradicate *L. monocytogenes* (3 log_10_ reduction in viability) at a very low concentration of 0.5 *μ*g/mL after a 10 min light irradiation (with a tungsten halogen lamp giving the power output of 165 mW) [54]. Moreover, at higher MB concentrations (up to 1 *μ*g/mL), the number of viable cells was decreased by up to 7 log_10_ cfu/mL. To inactivate *Candida *species,Queiroga et al. studied much higher concentrations of MB [55]. The PDT effect was strongest in the presence of 150 *μ*g/mL MB (78% reduction of CFU/mL) with a light dose of 180 J/cm^2^ (diode laser InGaAlP, 660 nm). To obtain this light dose, a longer irradiation time was necessary for lower light doses, and the authors suggested that therapy application time should be considered as an important factor. Because APDT is related to the production of toxic radicals such as singlet oxygen, the quantity of toxic radicals that are generated should increase as the irradiation time increases [55]. However, our results show no such correlation; for photodynamic inactivation, the light dose is important, not the irradiation time. We obtained the same results for the eradication of *S. aureus* with a light dose of 12 J/cm^2^, whether the irradiation time was 10 or 60 min (data not shown). To inactivate clinical isolates of *Staphylococcus* species, Miyabe et al. used 3 mM MB and a light fluence of 26.3 J/cm^2^ (gallium-aluminum-arsenide laser, 660 nm) to obtain a mean reduction of 6.29 log_10_ cfu/mL [56]. In *S. mutans,* Rolim et al. did not observe photodynamic activity when MB was used at a concentration of 163.5 *μ*M at 24 J/cm^2^ (LED, 640 nm), but a significant reduction (3 log_10_ cfu/mL) was observed when the same concentration of TBO and an equal light dose were used [57]. Maisch et al. reported that incubation of methicillin-sensitive *S. aureus* (MSSA), methicillin-resistant *S. aureus* (MRSA), *E. coli*, and *B. atrophaeus* with a porphyrin derivative (TMPyP) caused a biologically relevant decrease in CFU/mL upon illumination with multiple light flashes [58]. For MSSA, a TMPyP concentration of 1 *μ*M exhibited a killing efficacy of 2 log_10_ units reduction (at a radiant exposure of 80 J/cm^2^), and higher concentrations of TMPyP (10 or 100 *μ*M) caused a further decrease in bacterial survival (more than 5 log_10_ units). *E. coli* was decreased by 3 log_10_ cfu/mL units after photosensitization with 100 *μ*M TMPyP and a radiant exposure of 20 J/cm^2^ and by 5 log_10_ units after a radiant exposure of 40 J/cm^2^. However, concentrations of less than 100 *μ*M TMPyP did not induce photodynamic inactivation of *E. coli*, even with a radiant exposure of up to 80 J/cm^2^. MRSA strains that were photosensitized with TMPyP and illuminated under identical conditions exhibited a similar decrease in CFU/mL as that observed for the MSSA strain, indicating that the growth reduction was not dependent on the antibiotic resistance pattern. *B. atrophaeus* growth was reduced by more than 4 log_10_ by 10 *μ*M TMPyP and a single light flash of 10 or 20 J/cm^2^. For all of the studied strains, higher applied radiant exposures (up to 80 J/cm^2^) did not further increase the reduction in growth, and the authors suggested that increasing the radiant exposure appeared to produce a plateau in the killing efficacy [58]. To inactivate *P. chrysogenum* conidia, Gomes et al. studied porphyrin derivatives based on 5-,10-,15-,20-tetrakis(4-pyridyl) porphyrin and 5-,10-,15-,20-tetrakis(pentafluorophenyl) porphyrin [59]. A 4 log_10_ unit reduction was observed in the presence of 50 mM 5-,10-,15-,20-tetrakis(N-methylpyridinium-4-yl) porphyrin tetraiodide after 20 min of irradiation (white light at the fluence rate of 200 mW/cm^2^). Experiments performed with 100 mM 5-,10-,15-,20-tetrakis(N-methylpyridinium-4-yl) porphyrin tetraiodide and the additional step of removing the PS from the solution by centrifugation did not demonstrate an improvement in the photoinactivation efficiency [59]. Huang et al. studied the effects of PDT on Gram-positive bacteria (*S. aureus*), two different Gram-negative bacteria (*E. coli *and* P. aeruginosa*), and a fungal yeast (*C. albicans*) [60]. They used fullerene derivatives and white light to illuminate the cells and were able to reduce the growth of all tested microorganisms by 3 to 5 log_10_ units, depending on the microorganism and fullerene derivative. The most efficient was the BF2 derivative [60]. Park et al. demonstrated that chlorin Ce6-mediated PDT significantly reduced the colony formation of *S. aureus* in a dose-dependent manner [61]. Based on these data, it is clear that APDT can effectively kill various microbial species growing in planktonic culture.

#### 3.1.2. Biofilm Culture of Microorganisms

It is now well known that infections are mainly associated with biofilm formation. Collins et al. studied the effect of TMP on *P. aeruginosa* biofilms [62]. A significant decrease in biofilm density was observed, and the majority of the cells within the biofilm were nonviable when 100 *μ*M TMP and 10 min of irradiation (mercury vapor lamp, 220–240 J/cm^2^) were used. Moreover, the use of 225 *μ*M TMP and the same light dose resulted in almost complete disruption and clearance of the studied biofilm [62]. The effect of ZnPc-mediated APDT on yeast biofilms (*C. albicans*, non-*albicans Candida* species and non-*Candida* species) was studied by Junqueira et al. [63]. A gallium-aluminum-arsenide (GaAlAs) laser was used as the light source with the photosensitizer ZnPc at a concentration of 0.25 mg/mL. In all of the studied species, APDT caused reductions in CFU/mL values compared to the control group, but the levels of reduction ranged from 0.33 to 0.85 log_10_ for the various fungal species. The *Candida *spp. that were most resistant to APDT were *C. albicans*, *C. glabrata*, *C. norvegensis*, *C. krusei*, and *C. lusitaniae* (reduction <0.5 log_10_). The non-*Candida* pathogens *T. mucoides* and *K. ohmeri* were inactivated by APDT, with reductions of 0.85 log_10_ and 0.84 log_10_, respectively [63]. Biel et al. demonstrated that MB-mediated APDT is highly effective in the photoeradication of multispecies bacterial biofilms (multidrug-resistant *P. aeruginosa* and MRSA) [64, 65]. A significant decrease in CFU/mL (>6 log_10_ units) was achieved when 300 *μ*g/mL MB and a light dose of 60 J/cm^2^ (diode laser, 664 nm) were used. The reduction was >7 log_10_ units when 500 *μ*g/mL MB and two light doses of 55 J/cm^2^ separated by a 5-minute break were used [64, 65]. Meire et al. observed a statistically significant 1.9 log_10_ reduction in the viable counts of *E. faecalis* biofilms treated with 10 mg/mL MB and exposed to a soft laser of an output power of 75 mW (660 nm) for 2 min [66]. 

For biofilm-based cultures, much higher PS concentrations are required to obtain an APDT killing efficiency comparable to that observed for planktonic cultures. These higher concentrations may be potentially toxic for eukaryotic cells. Thus, it is of great importance to propose a strategy to decrease the PS concentrations used *in vivo* to further facilitate the application of APDT for the treatment of infections in humans and animals. Light parameters such as total light dose, beside the PS concentration, play an important role in APDT efficacy. In general, photoinactivation of microbial cells is dependent on light dose delivered to the sample and its efficacy is increasing with increasing light dose, considering particular light source of specific power density. In fact, lower PS concentration can be substituted by higher light doses, thus giving good opportunity to improve selectivity of APDT in potential clinical applications. The complexity of biological effects of irradiation of microbial cells as well as molecular responses to a PS itself (light-independent effects) demand individual optimization protocols for each reaction. 

### 3.2. Recent *In Vivo* Studies

Because APDT is an alternative and promising method for treating patients, *in vivo* studies are being conducted. Park et al. performed experiments on biofilms in an *in vivo* mouse model [61]. They demonstrated that Ce6-PDT treatment significantly reduced biofilm formation by *S. aureus* when treated with 10 *μ*M Ce6 and 10 J/cm^2^ of laser light. Because the *S. aureus* strain used in the study is bioluminescent, a bioluminescent *in vivo* imaging system (IVIS) was used. The group examined the effect of Ce6-mediated PDT on *in vivo* bacterial growth in a mouse model of skin infection with *S. aureus*, and the reduction in the intensity of bioluminescence was observed immediately after PDT. Moreover, on the 5th day after infection, the signal was almost undetectable in mice treated with Ce6-mediated PDT [61]. Dai et al. reported that a new MB-mediated APDT effectively treated *C. albicans* skin abrasion infections in mice [67]. In that study, a combination of 400 *μ*M NMB and 78 J/cm^2^ red light (Luma-Care lamp) was used to perform APDT 30 min after fungal inoculation, which resulted in a significant decrease in fungal luminescence (only few pixels corresponding to microbes could be observed immediately after APDT). Moreover, no significant reoccurrence of infection was observed at 24 h after APDT [67]. In an *in vivo* study by Hashimoto et al., APDT with 10 *μ*M hypocrellin B with lanthanide ions (HB:La^+3^) and a light dose of 24 J/cm^2^ (blue and red LED) reduced the number of *P. aeruginosa* in burn wounds, delaying bacteremia and decreasing bacterial levels in blood by 2-3 log_10_ compared to an untreated group [68]. Moreover, mice survival was increased at 24 h [68]. Fullerene-mediated APDT against *P. mirabilis* and *P. aeruginosa* wound infection was investigated by Lu et al. [69]. For *P. mirabilis* infection, 1 mM fullerenes (B6) and illumination with white light yielded a reduction of 96% after 180 J/cm^2^, which resulted in a highly significant increase in mouse survival of 82% [69]. For *P. aeruginosa*, the treatment gave a maximum reduction of 95%, but there was no beneficial effect on mouse survival (100% of the mice died within 3 days of infection) [69].

The infectious diseases that can be treated with APDT are mostly found in biofilm form, emphasizing the importance of focusing on the biofilm and its eradication, mass reduction, cell number reduction, and loss of viability. Photodynamic inactivation is a promising treatment option for eradication of microbial infections; however, as a biofilm treatment strategy, it has to overcome the obstacle of exopolymer matrix constituting a physical barrier for the photosensitizers as well as light.

## 4. Antibiofilm Strategies

Biofilm penetration by biocides or antibiotics is typically strongly hindered. To increase the efficiency of new treatment strategies against bacterial and fungal infections, factors that lead to biofilm growth inhibition, biofilm disruption, or biofilm eradication are being sought. These factors could include enzymes, sodium salts, metal nanoparticles, antibiotics, acids, chitosan derivatives, or plant extracts. All of these factors influence biofilm structure via various mechanisms and with different efficiencies.

### 4.1. Plant Extracts

 Numerous plants are used in folk medicine against various diseases. The increasing antibiotic resistance of pathogenic bacteria has resulted in increased attention by scientists to ethnopharmacology and alternative therapeutic options. Coenye et al. investigated five plant extracts with antibiofilm activity. Sub-MIC concentrations of *Rhodiola crenulata* (arctic root), *Epimedium brevicornum *(rowdy lamb herb), and *Polygonum cuspidatum *(Japanese knotweed) extracts inhibited *Propionibacterium acnes* biofilm formation by 64.8%, 98.5%, and 99.2%, respectively [70]. Moreover, active compounds within the extracts were identified and tested against three *P. acnes* strains. The most effective compound was resveratrol from *P. cuspidatum*, which reduced biofilm formation by 80% for each strain at a concentration of 0.32% (w/v). Icariin extracted from *E. brevicornum* reduced biofilm formation by 40%–70% at concentrations of 0.01%–0.08% (w/v). The antibiofilm activity of salidroside (0.02%–0.25% concentration) extracted from *R. crenulata* was strain dependent and yielded a biofilm reduction of 40% for *P. acnes* LMG 16711 and less than 20% for other tested strains.

*Melia dubia* (bead tree) bark extracts were examined by Ravichandiran et al.; at a concentration of 30 mg/mL, these extracts reduced *E. coli *biofilm formation by 84% and inhibited virulence factors such as hemolysins by 20% [71]. Bacterial swarming regulated by quorum sensing mechanisms (QS) was inhibited by 75%, resulting in decreased biofilm expansion [71]. Similar results were reported by Issac Abraham et al. concerning *Capparis spinosa *(caper bush) extract. At a concentration of 2 mg/mL, an inhibition of *E. coli* biofilm formation by 73% was observed [72]. For the pathogens *Serratia marcescens*, *P. aeruginosa,* and *P. mirabilis*, biofilm biomass was reduced by 79%, 75%, and 70%, respectively. Moreover, the mature biofilm structure was disrupted for all of the studied pathogens. Furthermore, the addition of *C. spinosa* extract (100 *μ*g/mL) to a bacterial culture resulted in swimming and swarming inhibition [72]. For *Lagerstroemia speciosa* (giant crape myrtle) extract, 83% biofilm inhibition was achieved at a concentration of 10 mg/mL [73]. Moreover, the anti-QS activity of the *L. speciosa* extract affected tolerance to tobramycin and reduced the expression of virulence factors such as LasA protease, LasB elastase, and pyoverdin [73].

The inhibition of biofilm formation is not the only antibiofilm strategy. Taganna et al. reported that a *Terminalia catappa* (bengal almond) extract at sub-MIC concentrations (500 *μ*g/mL and 1 mg/mL) stimulated biofilm formation; *P. aeruginosa* biofilm formation increased by 220% [74]. Despite increased biofilm formation, the *T. catappa* extract disrupted biofilm structure, and the administration of 1% SDS reduced the biofilm by 46%. Moreover, anti-QS activity and a 50% reduction of LasA expression were observed when the *T. catappa* extract was applied [74]. 

Highly effective antibiofilm activity was observed for fresh *Allium sativum* extract (fresh garlic extract, FGE). Fourfold treatment of a *P. aeruginosa* biofilm with FGE (24 hrs interval) resulted in biofilm reduction by 6 log_10_ units. Moreover, *in vivo* prophylactic treatment of a mouse model of kidney infection with FGE (35 mg/mL) for 14 days resulted in a 3 log_10_ unit decrease in the bacterial load on the fifth day after infection compared to untreated animals. In addition, FGE protected renal tissue from bacterial adherence and resulted in a milder inflammatory response and histopathological changes of infected tissues. Fresh garlic extract inhibited *P. aeruginosa* virulence factors such as pyoverdin, hemolysin, and phospholipase C. Moreover, killing efficacy and phagocytic uptake of bacteria by peritoneal macrophages were enhanced by garlic extract administration [75].

Extensive studies of the anti-*Staphylococcus epidermidis* biofilm activity of 45 aqueous extracts were published by Trentin et al. [76]. At 4 mg/mL, the most effective were extracts derived from *Bauhinia acuruana* branches (orchid tree), *Chamaecrista desvauxii* fruits, *B. acuruana* fruits, and *Pityrocarpa moniliformis* leaves, which decreased biofilm formation by 81.7%, 87.4%, 77.8%, and 77%, respectively. When applied at 10-fold lower concentration, noteworthy biofilm inhibition was observed only in the presence of *Commiphora leptophloeos* stem bark (corkwood) and *Senna macranthera* fruit extracts (reductions of 67.3% and 66.7%, resp.) [76].

Next, Carneiro et al. [77] tested sub-MIC concentrations of casbane diterpene (CS) extracted from *Croton nepetaefolius* bark against two Gram-positive bacteria (*S. aureus* and *S. epidermidis*), five Gram-negative bacteria (*Pseudomonas fluorescens*, *P. aeruginosa*, *Klebsiella oxytoca*, *K. pneumoniae*, and *E. coli*), and three yeasts (*Candida tropicalis*,* C. albicans,* and *C. glabrata*). *S. aureus* and *S. epidermidis* biofilms were significantly disrupted when CS was applied (125 *μ*g/mL and 250 *μ*g/mL, resp.). Among Gram-negative bacteria, *K. oxytoca *biofilms formation were not affected by CS, and *K. pneumoniae* biofilms were reduced by 45%. Administration of CS at a concentration of 125 *μ*g/mL caused complete inhibition of *P. fluorescens *biofilms (by 80%). However, lower concentrations of CS supported *P. aeruginosa* biofilm formation. Similar results were obtained for *E. coli*. The authors explained the observed phenomena by the enhanced production of exopolysaccharides due to the stress induced by the presence of CS in the culture. Casbane diterpene activity against *C. albicans* and *C. tropicalis* was observed, reducing biofilm formation by 50% (at concentrations of 62.5 *μ*g/mL and 15.6 *μ*g/mL, resp.) [77]. *Candida* biofilm formation was inhibited more effectively by *Boesenbergia pandurata* (fingerroot) oil [78]; biofilms were reduced by 63% to 98% when sub-MIC volumes (from 4 *μ*L/mL to 32 *μ*L/mL) were used. Moreover, a significant disruption of mature biofilms was observed when similar volumes of the tested oil were applied [78].

These data confirm that plant extracts have anti-QS, antiseptic, and antivirulence factor properties and can easily inhibit biofilm formation as well as disrupt the mature biofilm structure. Thus, plant extracts in combination with other antimicrobial strategies such as antibiotics or photodynamic inactivation could provide an effective bactericidal tool for the treatment of various bacterial and yeast infections.

### 4.2. Biofilm-Disrupting Enzymes

Because the biofilm matrix is composed of DNA, proteins, and extracellular polysaccharides, recent studies have indicated that the disruption of the biofilm structure could be achieved via the degradation of individual biofilm compounds by various enzymes.

#### 4.2.1. Deoxyribonuclease I

Tetz et al. [79] reported a strong negative impact of deoxyribonuclease I (DNase I) on the structures of biofilms formed by *Acinetobacter baumannii*, *Haemophilus influenzae*, *K. pneumoniae*, *E. coli*, *P. aeruginosa*, *S. aureus*, and *Streptococcus pyogenes*. Using DNase I at a concentration of 10 *μ*g/mL, degradation of mature 24 h formed biofilms by 53.85%, 52.83%, 50.24%, 53.61%, 51.64%, 47.65%, and 49.52%, respectively, was observed. Moreover, bacterial susceptibility to selected antibiotics increased in the presence of DNase I. Azithromycin, rifampin, levofloxacin, ampicillin, and cefotaxime were more effective in the presence of DNase I (5 *μ*g/mL) [79]. 

Furthermore, Hall-Stoodley et al. [80] reported that DNase I induced biofilm degradation by 66.7%–95% for six clinical isolates of *Streptococcus pneumoniae*, even though the biofilms were grown for six days. The authors revealed that the average biofilm thickness was reduced by 85%–97%, indicating that, within the biofilm, areas composed of lower amounts of extracellular DNA in comparison to adherent cells exist [80].

Moreover, Eckhart et al. [81] investigated the use of DNase I and DNase 1L2 (20 *μ*g/mL) against *S. aureus* and *P. aeruginosa* biofilms. Both enzymes revealed strong antibiofilm activity. After 7 hrs of incubation, *P. aeruginosa* biofilm formation was effectively reduced by DNase 1L2 treatment. However, eighteen hours of incubation in the presence of each enzyme resulted in weak inhibition of biofilm formation. *S. aureus* biofilm formation was significantly reduced by both enzymes, independent of the incubation time [81].

Furthermore, the antibiofilm activity of deoxyribonuclease I (130 *μ*g/mL) in combination with selected antibiotics toward *C. albicans* biofilms was estimated. A reduction of viable counts by 0.5 log_10_ units was observed for biofilm-growing *C. albicans* incubated with DNase I. Treating *C. albicans* with amphotericin B alone (1 *μ*g/mL) resulted in a 1 log_10_ unit reduction in cell viability, which increased to 3.5 log_10_ units in combination with DNase I. At higher concentrations of amphotericin B (>2 *μ*g/mL) and DNase I, cell viability was reduced by 5 log_10_ units. However, the fungicidal effectiveness of caspofungin and fluconazole decreased when combined with DNase I, indicating that the synergistic effect between the antibiotic and DNase I is dependent on the fungicidal agent used [82].

#### 4.2.2. Lysostaphin

Promising antibiofilm results were also obtained for lysostaphin. Lysostaphin is a natural staphylococcal endopeptidase that can penetrate bacterial biofilms [83, 84]. The antimicrobial properties of lysostaphin were analyzed by Walencka et al. [85], who reported the biofilm inhibitory concentration (BIC) of the enzyme for 13 *S. aureus* and 12 *S. epidermidis* clinical strains. The BIC against 8 *S. aureus* strains was estimated to be between 4 and 32 *μ*g/mL, and for the remaining 5 strains, the BIC value was higher than the maximum tested concentration (>64 *μ*g/mL). The majority of the studied *S. epidermidis *strains were more resistant to lysostaphin activity than were the *S. aureus* strains. Only 2 of the 12 *S. epidermidis* strains exhibited reduced biofilm formation in the presence of 128 *μ*g/mL or 16 *μ*g/mL lysostaphin. For the remaining 10 strains, the BIC value was estimated to be greater than 254 *μ*g/mL. In addition, the combined use of lysostaphin with oxacillin increased the susceptibility of the biofilm-growing bacteria to the antibiotic. However, no antibiofilm efficiency was observed for hetero-vancomycin-intermediate *S. aureus* and methicillin-resistant *S. epidermidis* strains [85].

High antibiofilm effectiveness of lysostaphin toward *S. aureus* strains was confirmed by Kokai-Kun et al. [86], who used a mouse model to determine the most effective treatment strategy for multiorgan biofilm infection. *S. aureus* biofilms, including methicillin-resistant *S. aureus* (MRSA), were completely eradicated in the presence of lysostaphin when animals were treated with the 15 mg/kg lysostaphin and 50 mg/kg of nafcillin, administered 3 times per day for four days. Moreover, lysostaphin (10 mg/kg) effectively protected indwelling catheters from bacterial infection [86].

In addition, Aguinaga et al. [87] reported that lysostaphin leads to significantly increased antibiotic susceptibility, with strain-dependent activity. The minimal biofilm eradication concentration (MBEC) for MRSA and MSSA strains was estimated for 10 antibiotics in combination with 20 *μ*g/mL lysostaphin. The highest synergistic effect was observed when lysostaphin was combined with doxycycline (MBEC decreased from 4 mg/mL to 0.5 mg/mL) or levofloxacin (MBEC decreased from 2 mg/mL to <1.9 mg/mL) against MRSA and MSSA, respectively [87].

#### 4.2.3. *α*-Amylases

Craigen et al. [88] analyzed the antibiofilm activity of *α*-amylases against strains of *S. aureus* and *S. epidermidis*. The tested enzymes effectively reduced formed biofilm and decreased biofilm formation in the case of *S. aureus*. However, no antibiofilm effect of the analyzed enzymes was observed for *S. epidermidis*. Time-course experiments for *S. aureus* showed that biofilms were degraded by 79% within 5 min and by 89% within 30 min of incubation with *α*-amylases. Amylase at doses of 10, 20, and 100 mg/mL reduced biofilms by 72%, 89%, and 90%, respectively, and inhibited matrix formation by 82%. In fact, *S. aureus* clinical isolates exhibited strain-dependent responses to amylase, but the treatment was successful for each strain. In addition, the antibiofilm activities of amylases from different biological sources were evaluated. The most effective biofilm reduction was reported for *α*-amylase isolated from *Bacillus subtilis*. Although enzymes derived from human saliva and sweet potato had no effect against preformed biofilms, all of the tested enzymes, regardless of origin, were highly effective in inhibiting biofilm formation [88].

#### 4.2.4. Lyase

Biofilms of two mucoid *P. aeruginosa* strains were treated with gentamycin (64 *μ*g/mL) in combination with alginate lyase (20 U/mL). The studied enzyme caused biofilm matrix liquefaction. Incubation of the biofilm with lyase and gentamycin for 96 h resulted in the complete eradication of the biofilm structure and living bacteria. A reduction of viable counts by 2-3 log_10_ units was reported for both strains when the combined therapy was applied [89].

#### 4.2.5. Lactonase

Kiran et al. [90] identified lactonase as a potential antibiofilm agent. Biofilms formed by 4 *P. aeruginosa* strains exhibited growth inhibition of 68.8%–76.8% in the presence of enzyme (1 U/mL) compared to the control sample. Moreover, 0.3 U/mL of the enzyme disrupted the biofilm structure and led to increased ciprofloxacin and gentamycin penetration and antimicrobial activity. Additionally, lactonase significantly decreased *P. aeruginosa* virulence factors such as pyocyanin (by 85%–93%), protease activity (by 86%–95%), elastase activity (by 69%–91%), and pyochelin secretion (by 40%–90%) [90].

### 4.3. Silver Nanoparticles

Silver is a nontoxic antimicrobial metal that can be used in medicine. Kalishwaralal et al. [91] analyzed the antibiofilm activity of silver nanoparticles (AgNPs) against *P. aeruginosa *and *S. epidermidis *strains. Nanoparticles were synthesized with *Bacillus licheniformis* and AgNO_3_. The mean diameter of the received particles was 50 nm. Incubation with AgNO_3_-containing nanoparticles (100 nM) inhibited the amount of biofilm formed after 24 h by 98%. Incubation with 50 nM AgNPs reduced exopolysaccharide content, indicating that biofilm formation was inhibited, although bacterial viability was unaffected [91].

Next, Mohanty et al. [92] reported dose-dependent antibiofilm activity of AgNPs against *S. aureus* and *P. aeruginosa*. Silver nanoparticles were prepared in 1% soluble starch with an average particle size of 20 nm. Incubation of biofilms (24 hr incubation) in the presence of 1 *μ*M or 2 *μ*M silver nanoparticles yielded greater than 50% or 85% inhibition of biofilm formation, respectively. Prolonged (48 hr) treatment resulted in 65% and 88% reduction of biofilm formation, respectively. A silver nanoparticle concentration of 0.1 *μ*M did not affect biofilm growth. Moreover, no significant cytotoxic effect was observed at any of the concentrations tested [92].

AgNP activity against *C. albicans* and *C. glabrata* biofilm formation was also estimated. Addition of silver nanoparticles to cultures of *Candida* adherent cells at a concentration of 3.3 *μ*g/mL reduced the percentage of total biomass of adherent *C. glabrata* cells by >90%. Moreover, mature biofilms after the treatment were significantly disrupted (97%) by 54 *μ*g/mL AgNPs. *C. albicans* biofilms exhibited increased resistance in comparison to *C. glabrata *with silver nanoparticle treatment, and an 85% reduction of adherent cell growth was observed at concentration >6.7 *μ*g/mL. No effect on mature biofilms was reported [93]. 

Chitosan-based silver nanoparticles (CS-AgNPs) reduced *P. aeruginosa* 24 hrs-grown biofilms by >65% at a concentration of 2 *μ*g/mL. *S. aureus* biofilms formation were inhibited by 22% by the same concentration of CS-AgNPs. Treatment with higher dose (5 *μ*g/mL) reduced biofilm formation by 65%. Scanning electron microscopy confirmed the destruction of the *P. aeruginosa* cell membrane by 2 *μ*g/mL CS-AgNPs. In addition, no cytotoxic effects toward macrophages were observed [94].

### 4.4. Other Biofilm-Disrupting Factors

As biofilm-related infections have become an increasingly prevalent problem in contemporary medicine, factors that disrupt biofilm structure or exhibit antibiofilm activity have been the subject of intense interest. 

The activities of three therapeutic molecules have been evaluated against *E. coli* biofilm formation. At concentrations of 30–125 *μ*g/mL, N-acetyl-L-cysteine reduced biofilm formation by 19.6%–39.7% for 5 of 7 *E. coli* strains. Ibuprofen exhibited greater efficacy, reducing biofilm formation by 37.2% to 44.8% (2–125 *μ*g/mL). Human serum albumin efficiently inhibited biofilm formation at the minimal tested concentration, 8 *μ*g/mL, reducing biofilm formation by 44.9%–79.4% [95].

Arias-Moliz et al. [96] investigated lactic acid at concentrations of 2.5%–20% and demonstrated its antimicrobial activity toward *E. faecalis* and *Enterococcus duran* strains. Complete eradication of biofilms was observed when 15% lactic acid was used for 1 min. In addition, 5% lactic acid reduced the viable cell count by 40.7%–100%. Simultaneous administration with 2% chlorhexidine slightly improved the killing efficacy of lactic acid, while administration with 0.2% cetrimide completely eliminated every tested strain independent of the lactic acid concentration used [96].

Chitosan also exhibits antibiofilm properties [97]. Chitosan nanoparticles were analyzed against 24 hour-formed biofilms of *S. mutans*. The antimicrobial effect of chitosan was tested against the three biofilm layers that could be identified within the mature biofilm structure: the upper (20 *μ*m), middle (15 *μ*m), and lower (2 *μ*m) biofilm layers. High-molecular-weight chitosan displayed biofilm reductions of 21.4% (upper layer), 7.5% (middle layer), and 1.2% (low layer). Low-molecular-weight chitosan reduced 24 hrs-formed biofilms by 93.6%–96.7% in each biofilm layer [97].

Furthermore, Orgaz et al. [98] analyzed the antibiofilm effectiveness of chitosan toward mature biofilms formed by *L. monocytogenes*, *Bacillus cereus*, *S. aureus*, *Salmonella enterica,* and *P. fluorescens*. The *Listeria* biofilm matrix was reduced by >6 log_10_, 4 log_10_, and 2.5 log_10_ units in the presence of 1%, 0.1%, and 0.01% chitosan, respectively. *P. fluorescens* exhibited 5 log_10_, 1.5 log_10_, and 1 log_10_ unit reductions, respectively, in the presence of identical concentrations of chitosan. For *Salmonella* and *Bacillus* species, a greater than 3 log_10_ unit reduction was not achieved (1% chitosan). The lowest antibiofilm effectiveness (1-2 log_10_ unit reduction) was obtained for *S. aureus *[98].

Recently, Sun et al. [99] reported the antibiofilm activity of terpinen-4-ol-loaded lipid nanoparticles against *C. albicans* biofilms. The compound used (10 *μ*g/mL) eradicated formed biofilms [99]. Finally, the antibiofilm activity of povidone-iodine (PVP-I) was confirmed by Hosaka et al. [100] against *Porphyromonas gingivalis *and *Fusobacterium nucleatum* biofilms. In the presence (5 min) of 7% PVP-I, 72 hour-formed biofilms of *P. gingivalis* exhibited a 6 log_10_ unit reduction in viable counts. Lower PVP-I concentrations (2%–5%) reduced biofilms by 2 log_10_ units. Biofilms formed by *F. nucleatum* were effectively reduced (by >4 log_10_) after 30 sec of incubation with 5% PVP-I [100].

Recently, numerous antibiofilm researches were published. Considering the fact, that various compounds acting against Gram-positive bacteria, Gram-negative bacteria, or fungi were analyzed, and different stage of biofilm growth (mature biofilm eradication or inhibition of biofilm formation) was assessed, it is difficult to reliably compare all the presented results. Some of the approaches seem, however, to be very promising. Among described plant extracts, fresh garlic showed the highest antibiofilm and antibacterial properties against *P. aeruginosa*. Also Japanese knotweed (*P. cuspidatum*) expresses good efficacy in the treatment of *P. acnes* biofilm formation. What seems, however, to be the most interesting is the ability to search for synergistic effects between different approaches, exemplified by the action of biofilm-disrupting enzymes in combination with antibiotics. *S. aureus* biofilm was completely disrupted by lysostaphin with nafcillin and *P. aeruginosa* biofilm by a combination of lyase with gentamycin, and DNaseI with amphotericin B effectively reduced *C. albicans* biofilm. Chitosan and chitosan-based silver nanoparticles can easily disrupt mature biofilm of *P. aeruginosa* and *S. mutans* and could provide penetration of biofilm structures by antimicrobials. This data suggested that APDT, enzymes, plant extracts, and other compounds can be used in various combinations acting as good antibiofilm and antimicrobial agents. The presented innovative strategies may potentially strongly support classical treatments and cause an increase of their effectiveness.

## 5. Conclusions 

In environments that include the human body, microbial cells form a well-organized structure termed a biofilm. The development of strategies to combat bacteria growing in biofilms is a challenging task; these bacteria are much more resistant to classical antimicrobial therapies and exchange genetic material more easily. Thus, under the pressure of a particular antibiotic, resistant clones are selected. Antimicrobial photodynamic therapy appears to be a very promising therapeutic option to effectively control the growth of microbial biofilms. However, as with other antimicrobial therapies, APDT is generally less effective against microorganisms growing in biofilms than against planktonic cells. Hence, there is a need to develop a therapeutic approach that would (i) increase the sensitivity of the microorganism to already established methods (e.g., antibiotic therapies) by violating the structure of the biofilm or disturbing the communication between a population of microorganisms in the biofilm or (ii) combine several modes of microbicidal action to achieve a synergistic effect. An example of the first approach is to use enzymes that affect the biofilm, while the second approach could be achieved by combining APDT with antibiotics, plant extracts, or biofilm-disrupting enzymes. Moreover, if we combine APDT with the use of enzymes that are specific for microbial structures; the selectivity of the approach will be increased as it potentially will permit the use of lower photosensitizer concentrations. One disadvantage of APDT is the limited amount of data based on animal models. However, the growing number of *in vivo* studies verifying APDT based on various photosensitizers is encouraging and will determine the direction of further research. 

## Figures and Tables

**Table 1:** APDT studies of planktonic microorganisms.

Microorganism	Photosensitizer	References
*Staphylococcus aureus,* *Escherichia coli,* *Pseudomonas aeruginosa,* and *Candida albicans *	Cationic fullerenes	Huang et al., 2010 [60]
*Penicillium chrysogenum *conidia	Cationic porphyrins	Gomes et al., 2011 [59]
*S. aureus *	Chlorin e6	Park et al., 2010 [61]
*Listeria monocytogenes *	MB	Lin et al., 2012 [54]
*Candida *spp.	MB	Queiroga et al., 2011 [55]
*Staphylococcus *spp.	MB	Miyabe et al., 2011 [56]
*Streptococcus mutans *	TBO, MB	Rolim et al., 2012 [57]
*Bacillus atrophaeus, *Methicillin-resistant *S. aureus * *Escherichia coli *	TMPyP (5-, 10-, 15-, 20-tetrakis (1-methylpyridinium-4-yl)-porphyrin tetra p-toluenesulfonate)	Maisch et al., 2012 [58]

**Table 2:** Recent APDT studies of biofilms and animal models.

Microorganism	Photosensitizer	Model	References
*P. aeruginosa, *Methicillin-resistant *S. aureus *	MB	Biofilm	Biel et al., 2011 [65]

*P. aeruginosa *	5-,10-,15-,20-tetrakis(1-methyl-pyridino)-21H, 23H-porphine, tetra-p-tosylate salt (TMP)	Biofilm	Collins et al., 2010 [62]

*Candida *spp.,* Trichosporon mucoides*, and* Kodamaea ohmeri *	Cationic nanoemulsion of zinc 2-,9-,16-,23-tetrakis(phenylthio)-29H, 31H-phthalocyanine (ZnPc)	Biofilm	Junqueira et al., 2012 [63]

*Enterococcus faecalis *	MB	Biofilm	Meire et al., 2012 [66]

*Proteus mirabilis* *P. aeruginosa *	Fullerenes B6	Mouse model	Lu et al., 2010 [69]

*C. albicans *	New MB	Mouse model	Dai et al., 2011 [67]

*S. aureus *	Chlorin e6	Mouse model	Park et al., 2010 [61]

*P. aeruginosa *	Hypocrellin B with lanthanide ions (HB:La^+3^)	Mouse model	Hashimoto et al., 2012 [68]
